# Aerobic and Muscle-Strengthening Physical Activity, Television Viewing, and Nonalcoholic Fatty Liver Disease: The CARDIA Study

**DOI:** 10.3390/jcm12175603

**Published:** 2023-08-28

**Authors:** Daniel J. McDonough, Mahesh Mathew, Zachary C. Pope, Pamela J. Schreiner, David R. Jacobs, Lisa B. VanWagner, John Jeffrey Carr, James G. Terry, Kelley Pettee Gabriel, Jared P. Reis, Mark A. Pereira

**Affiliations:** 1 Division of Epidemiology & Community Health, University of Minnesota-Twin Cities, Minneapolis, MN 55455, USA; mathe635@umn.edu (M.M.); schre012@umn.edu (P.J.S.); jacob004@umn.edu (D.R.J.J.); map@umn.edu (M.A.P.); 2 Well Living Lab, Rochester, NY 55902, USA; zachary.pope@delos.com; 3 Department of Physiology and Biomedical Engineering, Mayo Clinic, Rochester, NY 14625, USA; 4 Department of Internal Medicine, University of Texas Southwestern Medical Center, Dallas, TX 75390, USA; lisa.vanwagner@utsouthwestern.edu; 5 Vanderbilt University Medical Center, Vanderbilt University, Nashville, TN 37232, USA; j.jeffrey.carr@vumc.org (J.J.C.); james.g.terry@vumc.org (J.G.T.); 6 Department of Epidemiology, The University of Alabama at Birmingham, Birmingham, AL 35294, USA; gabrielk@uab.edu; 7 National Heart Lung and Blood Institute, Bethesda, MD 20892, USA; reisjp@nhlbi.nih.gov

**Keywords:** NAFLD, MASLD, cohort study, epidemiology, resistance exercise, sedentary behavior

## Abstract

Background: The prevalence of non-alcoholic fatty liver disease (NAFLD) in U.S. adults is over 30%, yet the role of lifestyle factors in the etiology of NAFLD remains understudied. We examined the associations of physical activity, by intensity and type, and television viewing with prevalent NAFLD. Methods: Cross-sectional analysis of a population-based sample of 2726 Black (49%) and White (51%) adults (Mean (SD) age, 50 (3.6) years; 57.3% female) from the CARDIA study. Exposures were aerobic activity by intensity (moderate, vigorous; hours/week); activity type (aerobic, muscle-strengthening; hours/week); and television viewing (hours/week), examined concurrently in all models and assessed by validated questionnaires. Our outcome was NAFLD (liver attenuation < 51 Hounsfield Units), measured by non-contrast computed tomography, after exclusions for other causes of liver fat. Covariates were sex, age, race, study center, education, diet quality, smoking status, alcohol consumption, and body mass index or waist circumference. Results: 648 participants had NAFLD. In the fully adjusted modified Poisson regression model, the risk ratios per interquartile range of each exposure were moderate-intensity aerobic activity, 1.10 (95% CI, 0.97–1.26); vigorous-intensity aerobic activity, 0.72 (0.63–0.82); muscle-strengthening activity, 0.89 (0.80–1.01); and television viewing, 1.20 (1.10–1.32). Relative to less active participants with higher levels of television viewing, those who participated in ≥2 h/week of both vigorous-intensity aerobic and muscle-strengthening activity and <7 h/week of television viewing had 65% lower risk of NAFLD (risk ratio = 0.35, 95% CI = 0.23–0.51). Conclusion: Adults who follow public health recommendations for vigorous-aerobic and muscle-strengthening activity, as well as minimize television viewing, are considerably less likely to have NAFLD than those who do not follow the recommendations and who have relatively high levels of television viewing.

## 1. Introduction

Nonalcoholic fatty liver disease (NAFLD)—defined as fat in the liver which is not due to excessive alcohol consumption, viral hepatitis, or medications—is the most prevalent liver disease in the world [1]. In the U.S., over 30% of adults have NAFLD [2] with an estimated 3.6 million new cases annually [3]. NAFLD is typically asymptomatic and clinically inconspicuous [2] despite portending a high risk for development of cardiometabolic diseases [4]. Preventing NAFLD and subsequent progression to more advanced liver disease (e.g., cirrhosis, hepatocellular cancer), cardiovascular disease, and mortality has become a public health priority given the burden on health care systems [5,6]. Considering that the pathogenesis of NAFLD is multifaceted [7] and some risk factors (e.g., genetics) are nonmodifiable, identifying its modifiable risk factors is of paramount importance. Given lifestyle modification is the cornerstone of treatment for NAFLD [2,8,9], examining lifestyle behaviors for primary prevention is warranted.

Regular physical activity participation is a modifiable lifestyle behavior that confers cardiometabolic health benefits (e.g., improved insulin sensitivity, modified hepatic de novo lipogenesis), which may protect against the metabolic drivers of NAFLD, independent of weight loss [10,11,12]. Higher physical activity levels have been observed to reduce free fatty acid uptake by the liver [13], and a growing body of evidence [14,15,16] indicates both aerobic and muscle-strengthening physical activity confer similar reductions in hepatic steatosis. Recent literature has suggested muscle-strengthening physical activity to be at least as efficacious as aerobic physical activity in the treatment of NAFLD [14,15,17]. Conversely, greater sedentary behavior (e.g., screen time) may catalyze adverse physiological adaptations that stimulate the metabolic underpinning of NAFLD (e.g., increased insulin resistance and lipogenesis) [18]. Although the independent associations of lower sedentary time and higher physical activity on health are not fully elucidated [19], reducing screen time is an emerging potential strategy, independent of physical activity, for reducing the burden of noncommunicable diseases [20]. Accordingly, epidemiological investigations are needed to more fully examine the roles of aerobic and muscle-strengthening physical activity, as well as screen time, in the etiology of NAFLD risk [21].

The current state of evidence regarding activity levels and NAFLD has largely focused on examining the association of aerobic and muscle-strengthening physical activity in reducing the negative health impact of hepatic steatosis. Thus, investigations examining the associations of aerobic and muscle-strengthening physical activity and screen time concurrently in the same models and the risk of developing NAFLD are needed to further examine the role of physical activity and sedentary time in the etiology of NAFLD. Therefore, in the Coronary Artery Risk Development in Young Adults (CARDIA) study, we concurrently examined the associations of aerobic physical activity (moderate- and vigorous-intensity), muscle-strengthening physical activity, and television viewing with the risk of NAFLD. We hypothesized that the risk of NAFLD would be lower with higher levels of moderate- and vigorous-intensity aerobic physical activity, muscle-strengthening physical activity, and lower levels of television viewing.

## 2. Materials and Methods

We adhered to the Strengthening the Reporting of Observational Studies in Epidemiology guidelines [22] for the drafting of this manuscript (Supplement Table S1).

### 2.1. Study Sample

CARDIA is an ongoing, multicenter, population-based cohort study of the development and determinants of cardiovascular disease in Black and White young adults (18–30 years, recruited from 1985 to 1986) across 4 U.S. cities (Birmingham, AL, USA; Chicago, IL, USA; Minneapolis, MN, USA; and Oakland, CA, USA). The study design has been described elsewhere [23]. Ten examinations have been completed to date, approximately every 2–5 years. Informed consent was obtained at each examination and the study was approved by the Institutional Review Boards at each CARDIA field center (University of Alabama Birmingham; Northwestern University; University of Minnesota; Kaiser Permanente). The present study includes participants who underwent computed tomographic (CT) scanning without contrast and have measures of liver steatosis obtained as part of the 25-year follow-up examination (Y25; 2010–11) [24,25].

**Figure 1:**
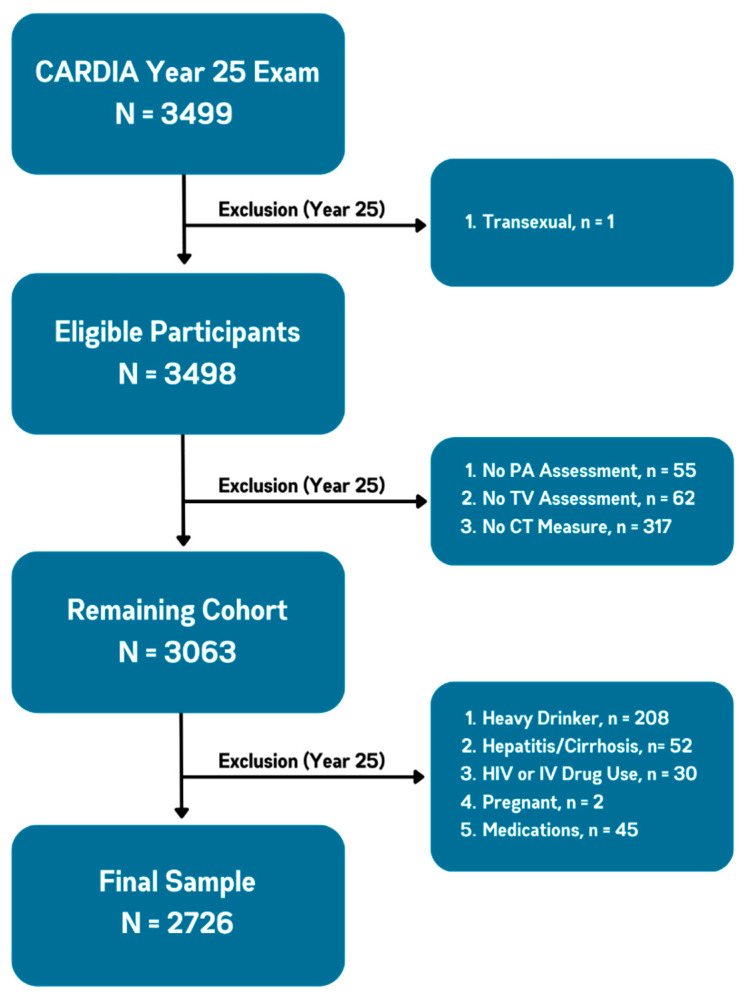
Participant Flow. Abbreviations: CARDIA, Coronary Artery Risk Development in Young Adults; PA, physical activity; TV, television viewing; CT, computed tomography; HIV, human immunodeficiency virus; IV, intravenous.

There were 3499 participants (46% men, 51% Black) who attended the Y25 examination. Participants were excluded from the CT exam if they weighed > 204 kg or were unable to fit within the CT gantry. We also excluded participants with missing measurements for physical activity, television viewing, or liver fat, as well as women who were pregnant, those with a self-reported history of hepatitis C or cirrhosis, a risk factor for chronic liver disease (e.g., intravenous drug use), or a potential cause of secondary hepatic steatosis (alcohol consumption ≥ 14 drinks/week in women and ≥21 drinks/week in men, self-reported human immunodeficiency virus, and medications known to cause hepatic steatosis (e.g., methotrexate, tamoxifen, amiodarone)). The remaining 2726 participants formed the sample population (Figure 1).

### 2.2. Measures

#### 2.2.1. Physical Activity and Television Viewing

Self-reported aerobic and muscle-strengthening physical activity were measured using the CARDIA Physical Activity History Questionnaire at the Y25 examination. Validity of the Questionnaire has been well established [26,27,28,29,30,31,32]. The Questionnaire asked about physical activity participation within the leisure-time and occupational domains over the previous 12 months in moderate-intensity aerobic physical activities (MPAs; nonstrenuous sports, walking, hiking, golfing, bowling, home exercises/calisthenics, and home maintenance or gardening) and vigorous-intensity aerobic physical activities (VPAs; running, jogging, racquet sports, biking, swimming, exercise/dance class, and strenuous sports). Muscle-strengthening physical activities included occupational lifting, carrying, or digging and leisure-time shoveling or weightlifting. Each activity was assigned a frequency based on whether it was performed for ≥1 h or during any 1 month in the past year, the number of months it was performed at that level, and the number of months it was performed on a physical activity-specific frequent basis. We report physical activity data in hours/week.

We measured television viewing using a questionnaire adapted from established sedentary behavior questionnaires [33,34]. Two studies using comparable questionnaires reported good reliability and fair-to-good validity, commensurate with other activity questionnaires [35,36]. The questionnaire measured non-work-related television viewing and participants could choose 1 of 9 potential durations with a minimum of ‘none’ and a maximum of ‘6 or more hours per day’. Participants reported usual weekday and weekend television viewing separately. We calculated television viewing as a weighted average of reported weekday and weekend time spent watching television. We report television viewing data in hours/week.

#### 2.2.2. Liver Attenuation

We measured liver attenuation in Hounsfield Units (HU) on 2.5 mm thick non-contrast CT images acquired using a multidetector 64-slice GE 750HD and LightSpeed VCT (GE Healthcare, Waukesha, WI, USA) at the Birmingham and Oakland centers, respectively, and a Siemens Sensation 64-slice (Siemens Medical Solutions, Erlangen, Germany) at the Chicago and Minneapolis centers. Our multi-center CT protocol and liver attenuation measurements have been described elsewhere [25,37,38]. Quality control and image analysis was performed at a core reading center (Wake Forest University Health Sciences, Winston-Salem, North Carolina). Measurement of liver attenuation was performed by trained readers in the right lobe of the liver using CT slices through the chest/thorax and was reported as the average of 9 measurements on 3 slices using circular regions of interest of 2.6 cm^2^, avoiding large vessels and lesions. The intraclass correlation coefficient between different readers on a blinded, randomly selected sample of 156 participants was 0.98 for liver attenuation, indicating high reproducibility of CT-measured liver attenuation in this study [39]. We used non-contrast CT-measured liver attenuation cut-offs of ≤40 HU (moderate-to-severe steatosis) and <51 HU (equivalent to liver-spleen ratio < 1; mild steatosis) to diagnose NAFLD when no other potential secondary causes of liver fat accumulation were present [40]. We report on <51 HU, which better captures NAFLD in an asymptomatic, population-based sample, while results for ≤40 HU are provided as a supplement.

### 2.3. Covariates

Trained CARDIA personnel administered standardized questionnaires to obtain demographic information on participant-identified race, sex, age, educational attainment (total years completed), smoking status (current, former, never), and alcohol consumption (drinks/week). Additionally, we used the a priori diet quality score from CARDIA Y20 to control for diet quality, with higher scores indicating higher quality diets [41]. Height (cm) and weight (kg) were measured in light clothing with a stadiometer and balance beam scale, respectively, and we calculated Y25 body mass index (BMI) as the weight in kilograms divided by the square of the height in meters. Waist circumference (cm) at Y25 was averaged over duplicate measurements by trained research personnel.

### 2.4. Statistical Analyses

SAS 9.4 (SAS institute, Cary, NC, USA) was used for all statistical analyses. We used modified Poisson regression models with robust standard errors to estimate the adjusted risk ratios (and their 95% confidence intervals) of NAFLD across each exposure: aerobic physical activity intensity (MPA and VPA); type of physical activity (i.e., aerobic and muscle-strengthening); and television viewing. In all analyses, all physical activity and television viewing exposure variables were included simultaneously in the same models. Our primary analysis examined all exposures continuously, standardized to their respective interquartile ranges (IQRs). To examine potential dose–response associations between the exposures and outcomes, we additionally constructed categorical models with the level of each exposure stratified into approximate quartiles. Model 1 was adjusted for sex, age, race, and CARDIA study center. Model 2 was additionally adjusted for education, diet, smoking status, and alcohol consumption. Models 3 and 3b were exploratory and were additionally adjusted for BMI and waist circumference in separate models to assess potential attenuation by general and central adiposity, respectively. Finally, we created a summary index of the combined levels of aerobic and muscle-strengthening physical activity and television viewing to describe how different combinations of the 3 exposures would be associated with risk of NAFLD. A summary of the 3 statistical analyses employed can be found in Table 1.

**Table 1:** Summary of statistical analyses.

Analysis	Model 1 Adjustments	Model 2 Adjustments	Model 3 Exploratory Adjustment	Model 3b Exploratory Adjustment
Table 2 Primary, Continuous Analysis	Age, race, sex, study center	Model 1 + diet quality, alcohol consumption, education, and smoking status	Model 2 + BMI	Model 2 + waist circumference
Table 3 Categorical Analysis to Elucidate Dose–response Associations	Age, race, sex, study center	Model 1 + diet quality, alcohol consumption, education, and smoking status	Model 2 + waist circumference	N/A. (BMI adjustment omitted given lack of attenuation of vigorous physical activity and television viewing in our primary analyses
Figure 2 Summary Index Model to Examine Additivity of the 3 Exposures	Adjusted for Model 2 (above). Not adjusted for exploratory adiposity confounders (BMI or waist circumference)

**Table 2:** Baseline characteristics among year 25 CARDIA participants, overall and by NAFLD category.

Variable	Overall (*n* = 2726)	Liver Attenuation	
		NAFLD <51 HU (*n* = 648)	No NAFLD ≥51 HU (*n* = 2078)
Liver Attenuation (HU), mean ± SD	55.6 ± 11.6	39.7 ± 10.9	60.6 ± 5.9
Age (years), mean ± SD	50.1 ± 3.6	50.3 ± 3.6	49.9 ± 3.6
Sex, *n* (%)			
Male	1163 (42.7)	359 (55.5)	804 (38.7)
Female	1562 (57.3)	288 (44.5)	1274 (61.3)
Race *n* (%)			
Black	1325 (48.6)	303 (46.8)	1022 (49.2)
White	1401 (51.4)	345 (53.2)	1056 (50.8)
Education (years), mean ± SD	15.1 ± 2.6	15.1 ± 2.7	14.9 ± 2.6
Diet Quality Score (std.), mean ± SD	5.4 ± 1.0	5.4 ± 1.0	5.3 ± 0.9
Smoking, *n* (%)			
Never	1717 (63.0)	373 (57.6)	1344 (64.7)
Former	590 (21.6)	167 (25.8)	423 (20.4)
Current	419 (15.4)	208 (16.7)	311 (15.0)
Alcohol (drinks/week), mean ± SD	7.4 ± 10.7	7.9 ± 11.7	7.3 ± 10.4
BMI (kg/m^2^), mean ± SD	30.4 ± 7.2	35.2 ± 7.3	28.9 ± 6.4
Waist Circumference (cm), mean ± SD	94.7 ± 15.8	108.4 ± 14.5	90.4 ± 13.6

Note: We excluded participants who were heavy drinkers (i.e., those who consumed ≥ 14 drinks/week) along with other factors related to liver disease (see Figure 1). Abbreviations: SD, standard deviation; NAFLD, nonalcoholic fatty liver disease; HU, Hounsfield Units; std., standardized score; BMI, body mass index.

**Table 3:** Adjusted risk of NAFLD per interquartile range of continuous physical activity and television viewing (hours per week).

Variable	Model 1	Model 2	Model 3	Model 3b
**Aerobic PA**				
Moderate (IQR = 4.33, h/wk)	1.08 (0.95–1.23)	1.10 (0.97–1.26)	1.14 (1.00–1.31)	1.16 (1.01–1.32)
Vigorous (IQR = 1.91, h/wk)	0.71 (0.63–0.81)	0.72 (0.63–0.82)	0.83 (0.73–0.94)	0.89 (0.79–1.02)
**Muscle-Strengthening PA** (IQR = 3.33, h/wk)	0.89 (0.79–1.00)	0.89 (0.80–1.01)	0.93 (0.83–1.05)	0.95 (0.84–1.07)
**Television Viewing** (IQR = 14.0, h/wk)	1.11 (1.05–1.18)	1.12 (1.05–1.19)	1.07 (1.01–1.14)	1.06 (0.99–1.14)

Note: Data displayed as risk ratios and 95% confidence intervals. All physical activity and television viewing variables are included simultaneously in all models. Model 1, adjusted for age, race, sex, study center; Model 2 additionally adjusted for diet quality, alcohol consumption, education, and smoking status; Model 3 additionally adjusted for body mass index (BMI); Model 3b additionally adjusted for waist circumference rather than BMI. Abbreviations: h/wk, hours per week; PA, physical activity; IQR, interquartile range.

## 3. Results

### 3.1. Participant Characteristics

The prevalence of NAFLD was 648/2726 (23.8%). Table 2 shows demographics and other participant characteristics stratified by NAFLD status. The mean (SD) age of the total sample was 50.1 (3.6) years, 1325 (49%) were Black, 1163 (43%) were male, and the mean (SD) years of education was 15.1 (2.6). Among participants with NAFLD, 359 (55.5%) were male and 303 (46.8%) were Black. Age, education level, weekly alcohol consumption, and diet quality were similar between those with and without NAFLD. Participants with NAFLD had greater general and central adiposity as measured using BMI and waist circumference, respectively.

### 3.2. Primary Analysis

Results for our primary analysis are presented in Table 3. After adjustment for age, race, sex, and study center, we observed a statistically significantly lower risk of NAFLD among participants who engaged in higher levels of VPA and lower levels of television viewing. We observed similar results after further adjusting for diet quality, weekly alcohol consumption, education, and smoking status in Model 2. Waist circumference, but not BMI, materially attenuated the risk ratios for VPA, muscle-strengthening physical activity, and television viewing. Muscle-strengthening physical activity was associated with lower risk of NAFLD in all models, but these findings did not reach statistical significance. Moderate-intensity aerobic physical activity was not associated with NAFLD. Findings from our sensitivity analyses for NAFLD ≤ 40 HU were consistent with our primary findings (Supplement Tables S2 and S3).

### 3.3. Categorical (Dose–Response) Analysis

Our categorical analysis with levels of physical activity intensity and television viewing stratified into quartiles is displayed in Table 4. We observed a dose–response association between VPA and risk of NAFLD. Relative to 0 h/week in Model 2, participation in 0.51–1.99 h/week of VPA was associated with 27% lower risk of NAFLD and ≥2 h/week was associated with 43% lower risk of NAFLD. Muscle-strengthening physical activity, relative to 0 h/week in Model 2, was also associated with lower risk of NAFLD, with evidence of a dose–response (13% lower risk for 0–1.99 h/week and 22% lower risk for 2–3.99 h/week). For television viewing, relative to <7 h/week in Model 2, risk of NAFLD increased by 38% for 7–13.99 h/week and by 83% for 14–20.99 h/week. All findings were attenuated with adjustment for waist circumference in Model 3b, with only television viewing remaining statistically significant at ≥21 h/week.

### 3.4. Summary Index

**Figure 2:**
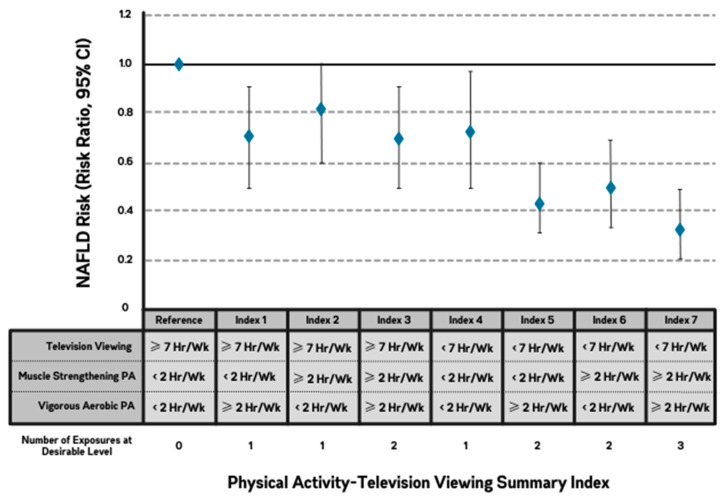
Physical Activity-Television Viewing Summary Index. Note: Thresholds for desirable physical activity exposures were ≥2 h/week of vigorous-aerobic and muscle-strengthening physical activity, based on recommendations from the 2020 World Health Organization Physical Activity Guidelines. The threshold for desirable television viewing was ≤7 h/week based on observed distribution. Moderate-intensity physical activity was omitted due to lack of statistical significance across all models in our primary and secondary analyses. The study adjusted for age, race, sex, study center, diet quality, alcohol consumption, education, and smoking status (and was not adjusted for exploratory adiposity confounders (body mass index or waist circumference)). Abbreviations: NAFLD, nonalcoholic fatty liver disease; PA, physical activity; Hr, hours; Wk, week; CI, confidence interval.

The physical activity-television viewing summary index (Figure 2) demonstrated the additive associations of VPA, muscle-strengthening physical activity, and television viewing with the risk of NAFLD. Specifically, relative to the lowest categories of VPA and muscle-strengthening physical activity and highest category of television viewing, concurrent higher levels of VPA (≥2 h/week), muscle-strengthening physical activity (≥2 h/week), and lower levels of television viewing (<7 h/week), were associated with 65% lower risk of NAFLD (risk ratio = 0.35, 95% CI = 0.23–0.51).

## 4. Discussion

To our knowledge, this is the first study to include physical activity, stratified by intensity and type, and television viewing in the same models to examine associations with the risk of NAFLD. Higher levels of VPA and muscle-strengthening physical activity and lower levels of television viewing were associated with lower risk of NAFLD in a dose–response manner. We observed additive associations among the three exposures, such that concurrent high levels of VPA and muscle-strengthening physical activity and low levels of television viewing reduced the risk of NAFLD by 65%. Adjustments for BMI and waist circumference were exploratory, and results should be interpreted with caution because these measures of adiposity are influenced by physical activity and television viewing and may be on the causal pathway to NAFLD. Adjustment for BMI did not materially alter the findings. However, waist circumference attenuated the risk ratios for physical activity and television viewing. Interestingly, MPA was not associated with NAFLD risk in any model.

Our findings are in line with previous human trials [10,11,12,14,15,16,17,42,43,44,45] and epidemiological evidence [21,46,47,48,49,50], suggesting that replacing sedentary time with aerobic and/or muscle-strengthening physical activity leads to attenuation of intrahepatic lipid deposition. Two studies specifically examined the associations of television viewing with the risk of NAFLD while accounting for physical activity and observed a dose-dependent increased risk of NAFLD with sedentary television viewing [51,52]. However, these studies only examined general leisure- and occupational-domain physical activities and did not examine the independent and additive role of muscle-strengthening physical activity—a physical activity modality that may provide various cardiometabolic health benefits independent of aerobic activity. Our study was the first to analyze physical activity by intensity (moderate, vigorous) and type (aerobic, muscle-strengthening) concurrently within the same models to determine whether lower sedentary time and/or higher aerobic and muscle-strengthening physical activity have anti-steatotic effects in the primary prevention of NAFLD. We hypothesized that both MPA and VPA are associated with a lower risk of NAFLD [45,53,54]. However, while higher VPA and muscle-strengthening physical activity and lower television viewing time were associated with a lower risk of NAFLD, MPA was not associated with a lower risk of NAFLD. Exploratory analyses demonstrated little to no attenuation of the risk ratios with adjustment for BMI, whereas waist circumference considerably attenuated the risk ratios. The findings for waist circumference adjustment were expected as increased physical activity and reduced sedentary behavior are known to reduce adiposity [55,56,57,58], and waist circumference may have a higher correlation with intra-abdominal adipose (including hepatic fat) than BMI [59].

Current evidence suggests the pathogenesis of NAFLD is largely driven by insulin resistance in the liver, skeletal muscle, and adipose tissue, resulting in peripheral lipolysis, increased delivery of free fatty acids to the liver, and hepatic de novo lipogenesis [7,60,61]. Regular aerobic and muscle-strengthening physical activity and reduced sedentary time protect against the preceding cardiometabolic drivers of NAFLD, independent of weight loss [13,62,63]. Aerobic physical activity may reduce the risk of NAFLD through various pathways (e.g., activation of lipolysis, upregulation of uncoupling protein-1, and alteration of adipocytokines) [14]. Vigorous-intensity aerobic physical activity, specifically, relies on glycolytic energy production, which accumulates lactate in the muscle and blood—an important metabolic substrate and signaling molecule that mediates exercise adaptations and inter-organ communication [64,65,66]. Similarly, the skeletal muscle acts as an endocrine organ [67], and muscle-strengthening physical activity produces myokines (e.g., interleukin-6, irisin) [68,69] that mediate muscle–liver crosstalk through which lipogenesis in hepatocytes may be inhibited [14]. Further, VPA and muscle-strengthening physical activity require contraction of the type II muscle fibers, potentiating fiber type-specific alterations, including the upregulation of glycolysis and downstream amelioration of insulin resistance [70,71]. Finally, muscle-strengthening physical-activity-mediated hypertrophy of the large, type II muscle fibers increases the available glucose storage area, further improving insulin sensitivity and maintenance of glucose homeostasis [72].

Obesity, particularly central obesity [59], is an important risk factor for NAFLD. Indeed, our exploratory analyses demonstrated waist circumference materially attenuated the associations between physical activity, television viewing, and NAFLD. In addition to the direct metabolic pathways described above, higher physical activity and lower television viewing may reduce the risk of NAFLD more generally through improved energy balance and body fat distribution. Interestingly, compared to VPA, MPA has been observed to elicit similar changes in anthropometric outcomes [55], yet a recent meta-analysis showed that VPA elicits greater fat oxidation during exercise [56]. Although exercise-related thermogenesis is negligible with regard to total daily energy expenditure in the general population [73], there is increased interest in VPA for weight reduction, given the ensuing recovery demand and oxygen debt [74]. While the acute increase in post-VPA caloric expenditure is minimal, the long-term effects of habitual VPA may be substantial [74]. Replacing sedentary time with 100 kilocalories per day of physical activity while maintaining a consistent energy intake has been proposed as a solution to prevent population-level weight gain [75] and may have an even more profound effect if specifically replaced with VPA. Finally, both VPA and muscle-strengthening physical activity have profound effects on reducing splanchnic fat and increasing lean body mass [57,58,76]. Given the increasing body of evidence suggesting MPA is beneficial for health [54,77], it may be that participants who preferentially performed MPA over VPA did not do so at adequate daily durations to produce meaningful changes in energy expenditure or intrahepatic fat.

### Strengths and Limitations

Our study has the following strengths: (1) CARDIA is a large, multi-center, prospective cohort study with a population-based sample of U.S. Black and White men and women; (2) we robustly characterized potential confounders, thereby minimizing bias from measured or residual confounding; and (3) we simultaneously modeled three types of physical activity and television viewing to address potential independence across these exposures, and tested dose–response associations with sufficient power to detect clinically meaningful associations.

Our results should be interpreted while considering the following limitations. First, this study was cross-sectional, and we cannot infer temporality or causation. However, the cross-sectional design was not likely a significant threat to validity because NAFLD is a silent disease (typically undiagnosed), thereby minimizing, but not ruling out, concerns of reverse causality. Second, despite robust adjustment for many covariates that may be confounders and/or on the causal pathway to minimize potential residual confounding, we observed some variability in our results. Future prospective study designs are needed to elucidate the potential impact of other cardiometabolic comorbidities and/or environmental factors on the interplay of the examined exposures and outcomes. Third, although we demonstrated robust inter-rater reliability for our multi-center CT protocol to determine NAFLD status [39], we did not perform concurrent laboratory risk stratification tests for fibrosis. Nevertheless, we measured and demonstrated the degree of steatosis using CT scans without contrast–a method with similar test performance to that of ultrasound-based measures (e.g., controlled attenuation parameter). Unfortunately, contemporaneous measurement of liver chemistries alongside hepatic steatosis ascertainment did not occur in CARDIA, limiting our ability to look at noninvasive markers of severity of steatosis (e.g., fatty liver index) or fibrosis (e.g., FIB-4 score). However, histologic diagnosis of the disease is more than minimal and not appropriate for steatosis ascertainment in the context of a large, prospective cohort study. Finally, our exposures (physical activity and television viewing) were assessed using self-report instruments, which are prone to recall and/or social desirability bias. Nevertheless, our physical activity and television viewing assessments have been directly and indirectly validated against multiple measures of habitual physical activity [26,27,28,29,30,31,32,35,36].

## 5. Conclusions

Our findings suggest that higher levels of VPA and muscle-strengthening physical activity and lower levels of television viewing are associated with lower risk of NAFLD in a dose–response manner. When compared to the lowest categories of physical activity and highest category of television viewing, higher levels of both physical activity types combined with lower levels of television viewing were associated with a 65% lower risk of NAFLD.

## Figures and Tables

**Table 4:** Adjusted risk of NAFLD per category of physical activity and television viewing.

Variable	Model 1	*^a^P*	Model 2	*^a^P*	Model 3b	*^a^P*
Aerobic PA						
Moderate (h/wk) ≤1.7, (*n* = 697) 1.71–3.8, (*n* = 682) 3.81–6, (*n* = 671) >6, (*n* = 676)	- 1.03 (0.83–1.30) 1.10 (0.87–1.30) 1.14 (0.89–1.45)	0.27	- 1.05 (0.83–1.32) 1.14 (0.90–1.44) 1.17 (0.91–1.51)	0.18	- 1.04 (0.82–1.31) 1.19 (0.94–1.51) 1.21 (0.94–1.57)	0.09
Vigorous (h/wk) 0, (*n* = 946) 0–0.5, (*n* = 456) 0.51–1.99, (*n* = 635) ≥2, (*n* = 676)	- 0.94 (0.75–1.17) 0.82 (0.66–1.01) 0.56 (0.44–0.71)	<0.001	- 0.96 (0.76–1.20) 0.83 (0.67–1.03) 0.57 (0.44–0.73)	<0.001	- 1.14 (0.90–1.43) 1.06 (0.85–1.33) 0.89 (0.69–1.16)	0.11
Muscle-Strengthening PA (h/wk) 0, (*n* = 840) 0–1.99, (*n* = 682) 2–3.99, (*n* = 671) ≥4.0, (*n* = 676)	- 0.87 (0.71–1.07) 0.78 (0.59–1.03) 0.80 (0.63–1.01)	0.04	- 0.87 (0.71–1.08) 0.78 (0.59–1.03) 0.81 (0.63–1.03)	0.04	- 0.99 (0.81–1.24) 0.92 (0.70–1.22) 0.95 (0.74–1.21)	0.57
Television Viewing (h/wk) <7, (*n* = 882) 7–13.99, (*n* = 750) 14–20.99, (*n* = 437) ≥21, (*n* = 574)	- 1.39 (1.12–1.73) 1.82 (1.43–2.32) 1.75 (1.37–2.24)	<0.001	- 1.38 (1.10–1.73) 1.83 (1.43–2.33) 1.75 (1.36–2.25)	<0.001	- 1.19 (0.95–1.49) 1.27 (0.99–1.63) 1.37 (1.07–1.77)	0.01

*^a^P* = linear trend significance level. Data displayed as risk ratios and 95% confidence intervals. Note: All Physical activity and television viewing variables are included simultaneously in all models. Model 3 BMI adjustment was omitted given lack of attenuation of vigorous physical activity and television viewing in our primary analyses (Table 3). Model 1 adjusted for age, race, sex, study center; Model 2 additionally adjusted for diet quality, alcohol consumption, education, and smoking status; Model 3b additionally adjusted for waist circumference. Abbreviations: h/wk, hours per week; PA, physical activity.

## Data Availability

No new data were created or analyzed in this study. Data sharing is not applicable to this article.

## Supplementary Materials

The following supporting information can be downloaded at: https://www.mdpi.com/article/10.3390/jcm12175603/s1. Table S1. STROBE statement—checklist for reports of cross-sectional studies. Table S2. Baseline characteristics among year 25 CARDIA participants, overall and by moderate-to-severe NAFLD category. Table S3. Sensitivity analysis of the adjusted risk of moderate-to-severe steatosis (NAFLD ≤ 40 Hounsfield Units) per interquartile range of continuous physical activity and television viewing (hours per week).
