# Supplementary material for: Aerobic and Muscle-Strengthening Physical Activity, Television Viewing, and Nonalcoholic Fatty Liver Disease: The CARDIA Study
Source: J Clin Med. 2023 Aug 28;12(17):5603. doi: 10.3390/jcm12175603 (PMC10488389; doi:10.3390/jcm12175603)
Supplement: Supplementary file 1 [file jcm-12-05603-s001.zip › jcm-2537444-supplementary.pdf]

Supplemental Content

McDonough DJ, Mathew M, Pope ZC, et al. Aerobic and muscle-strengthening physical activity, television viewing, and nonalcoholic fatty liver disease: the CARDIA study.

Table S1. STROBE statement — checklist for reports of cross-sectional studies.
Table S2. Baseline characteristics among year 25 CARDIA participants, overall and by moderate-to-severe NAFLD category.
Table S3. Sensitivity analysis of the adjusted risk of moderate-to-severe steatosis (NAFLD ≤40 Hounsfield Units) per interquartile range of continuous physical activity and television viewing (hours per week).

Table S1. STROBE statement — checklist for reports of cross-sectional studies.

	Item No	Recommendation	Page No
**Title and abstract**	1	(*a*) Indicate the study's design with a commonly used term in the title or the abstract	3-4
		(*b*) Provide in the abstract an informative and balanced summary of what was done and what was found	3-4
**Introduction**			
Background/rationale	2	Explain the scientific background and rationale for the investigation being reported	5-6
Objectives	3	State specific objectives, including any prespecified hypotheses	5-6
**Methods**			
Study design	4	Present key elements of study design early in the paper	7
Setting	5	Describe the setting, locations, and relevant dates, including periods of recruitment, exposure, follow-up, and data collection	7
Participants	6	(*a*) Give the eligibility criteria, and the sources and methods of selection of participants	7
Variables	7	Clearly define all outcomes, exposures, predictors, potential confounders, and effect modifiers. Give diagnostic criteria, if applicable	8-10
Data sources/ measurement	8	For each variable of interest, give sources of data and details of methods of assessment (measurement). Describe comparability of assessment methods if there is more than one group	8-10
Bias	9	Describe any efforts to address potential sources of bias	7-10
Study size	10	Explain how the study size was arrived at	7
Quantitative variables	11	Explain how quantitative variables were handled in the analyses. If applicable, describe which groupings were chosen and why	7-10
Statistical methods	12	(*a*) Describe all statistical methods, including those used to control for confounding	10
		(*b*) Describe any methods used to examine subgroups and interactions	10
		(*c*) Explain how missing data were addressed	10
		(*d*) If applicable, describe analytical methods taking account of sampling strategy	N/A
		(*e*) Describe any sensitivity analyses	10
**Results**			
Participants	13	(a) Report numbers of individuals at each stage of study—eg numbers potentially eligible, examined for eligibility, confirmed eligible, included in the study, completing follow-up, and analysed	11
		(b) Give reasons for non-participation at each stage	11
		(c) Consider use of a flow diagram	Fig.1
Descriptive data	14	(a) Give characteristics of study participants (eg demographic, clinical, social) and information on exposures and potential confounders	11
		(b) Indicate number of participants with missing data for each variable of interest	11
	**Item No**	**Recommendation**	**Page No**
Outcome data	15	Report numbers of outcome events or summary measures	11

Main results	16	(*a*) Give unadjusted estimates and, if applicable, confounder-adjusted estimates and their precision (eg, 95% confidence interval). Make clear which confounders were adjusted for and why they were included	11-12
		(*b*) Report category boundaries when continuous variables were categorized	11-12
		(*c*) If relevant, consider translating estimates of relative risk into absolute risk for a meaningful time period	N/A
Other analyses	17	Report other analyses done—eg analyses of subgroups and interactions, and sensitivity analyses	11-12
**Discussion**			
Key results	18	Summarise key results with reference to study objectives	13
Limitations	19	Discuss limitations of the study, taking into account sources of potential bias or imprecision. Discuss both direction and magnitude of any potential bias	16
Interpretation	20	Give a cautious overall interpretation of results considering objectives, limitations, multiplicity of analyses, results from similar studies, and other relevant evidence	13-16
Generalisability	21	Discuss the generalisability (external validity) of the study results	17
**Other information**			
Funding	22	Give the source of funding and the role of the funders for the present study and, if applicable, for the original study on which the present article is based	16-17

Table S2. Baseline characteristics among year 25 CARDIA participants, overall and by moderate-to-severe NAFLD category.

Variable	Overall (n = 2726)	Liver Attenuation	
		Moderate-to-Severe NAFLD ≤40 HU (n = 262)	No NAFLD >40 HU (n = 2464)
Liver Attenuation (HU), mean ± SD	55.6 ± 11.6	29.2 ± 9.9	58.5 ± 7.5
Age (years), mean ± SD	50.1 ± 3.6	50.5 ± 3.7	50.0 ± 3.6
Sex, *n* (%)			
Male	1163 (42.7)	144 (55.2)	1019 (41.4)
Female	1562 (57.3)	117 (44.8)	1445 (58.6)
Race *n* (%)			
Black	1325 (48.6)	113 (43.1)	1212 (49.2)
White	1401 (51.4)	149 (56.9)	1252 (50.8)
Education (years), mean ± SD	15.1 ± 2.6	15.0 ± 2.7	14.9 ± 2.6
Diet Quality Score (std.), mean ± SD	5.4 ± 1.0	5.4 ± 1.0	5.3 ± 0.9
Smoking, *n* (%)			
Never	1717 (63.0)	157 (59.9)	1560 (63.3)
Former	590 (21.6)	70 (26.7)	520 (21.1)
Current	419 (15.4)	35 (13.4)	384 (15.6)
Alcohol (drinks/week), mean ± SD	7.4 ± 10.7	7.3 ± 11.3	7.4 ± 10.7
BMI (kg/m^2), mean ± SD	30.4 ± 7.2	36.3 ± 7.4	29.8 ± 6.8
Waist Circumference (cm), mean ± SD	94.7 ± 15.8	112.1 ± 14.8	92.8 ± 14.8

Note. We excluded participants who were heavy drinkers (i.e., those who consumed ≥14 drinks/week) along with other factors related to liver disease (see Figure 1 in the main text). Abbreviations: SD, standard deviation; NAFLD, nonalcoholic fatty liver disease; HU, Hounsfield Units; std., standardized score; BMI, body mass index.

Table S3. Sensitivity analysis of the adjusted risk of moderate-to-severe steatosis (NAFLD ≤40 Hounsfield Units) per interquartile range of continuous physical activity and television viewing (hours per week).

Variable	Model 1	Model 2	Model 3	Model 3b
**Aerobic PA**				
Moderate (IQR = 4.33, hrs/wk)	1.04 (0.85-1.27)	1.08 (0.88-1.33)	1.14 (0.93-1.42)	1.17 (0.95-1.44)
Vigorous (IQR = 1.91, hrs/wk)	0.63 (0.51-0.78)	0.65 (0.52-0.81)	0.78 (0.63-0.97)	0.88 (0.71-1.09)
**Muscle-Strengthening PA** (IQR = 3.33, hrs/wk)	1.00 (0.83-1.20)	1.01 (0.84-1.21)	1.06 (0.88-1.28)	1.09 (0.90-1.32)
**Television Viewing** (IQR = 14.0, hrs/wk)	1.10 (1.00-1.21)	1.12 (1.02-1.24)	1.06 (1.01-1.18)	1.06 (0.94-1.19)

Note. Data displayed as risk ratios and 95% confidence intervals. All physical activity and television viewing variables are included simultaneously in all models. Model 1, adjusted for age, race, sex, study center; Model 2 additionally adjusted for diet quality, alcohol consumption, education, and smoking status; Model 3 additionally adjusted for body mass index (BMI); Model 3b additionally adjusted for waist circumference rather than BMI. Abbreviations: hrs/wk, hours per week; PA, physical activity. Abbreviations: hrs/wk, hours per week; PA, physical activity; IQR, interquartile range.
